# Ten public health strategies to control the Covid-19 pandemic: the Saudi Experience

**DOI:** 10.1016/j.ijregi.2021.09.003

**Published:** 2021-09-17

**Authors:** Areej AlFattani, Amani AlMeharish, Maliha Nasim, Khalid AlQahtani, Sami AlMudraa

**Affiliations:** 1 King Faisal Specialist Hospital and Research Center, Department of Biostatics, Epidemiology and Scientific Computing, Riyadh, Saudi Arabia; 2 Prince Sattam Bin Abdul Aziz University, college of science and humanities, Alkharj, Saudi Arabia; 3 Ministry of Health, Health Operation Center; 4 Saudi Epidemiology Society

**Keywords:** Covid_19, Saudi Arabia, Pandemic, experience, strategies, control, precautions

## Abstract

Saudi Arabia plays an important strategic role within the Middle East and afar because of its geographical location, and being the host of one of the largest annual religious mass gatherings in the world “The Hajj”. During the recent coronavirus pandemic, the Kingdom of Saudi Arabia (KSA) developed a multisectoral plan that adopted multiple measures to limit the spread of Covid-19 transmission both domestically and internationally. In this article, we review all public health related policy decisions from the Saudi Ministry of Health, other government departments, and the private sector that contributed to limiting the severe consequences from Covid-19. Ten effective strategies are outlined and the challenges related to their implementation are explored. The strategies include: 1. Quarantine and travel restriction, 2. Expansion of serological screening, 3. Mask wearing (covering the face and nose) and social distancing, 4. Preparation of hospitals to deal with the influx of coronavirus cases, 5. Use of artificial intelligence, 6.Public assurance, 7.Removal of slum areas and re housing of its inhabitants, 8. Cancellation of the Hajj season, 9. Economic stimulus packages to safeguard the economy, and 10. fair and priority driven vaccine distribution.

Conclusion: The government of Saudi Arabia demonstrated responsibility at the highest level to prioritize the safety and well-being of its citizens and residents. Rapid early response to the pandemic warnings, extensive experience in previous epidemics and in mass gathering medicine, wise management of healthcare resources, and unprecedented harmonization of governmental and private sectors were significant factors for this success.

## Highlights

• KSA plans during the pandemic in managing the healthcare system were robust and sustainable.
• Previous epidemics and mass gathering have helped to use the resources effectively.
• Digital health and the prompt response to pandemic warnings were factors of success
• Healthcare supplies and difficulties of online education were among the challenges.
• It is recommended to revise the disaster and emergency strategic plans in KSA.

## Introduction

It has been 20 months since the ongoing Covid-19 pandemic started in December 2019. The SARS-COV-2 virus is characterized by complexity, high transmissability through human-to-human infection, and a high percentage of asymptomatic carriers (Shereen et al., 2020). By September 2021, almost 222 million people had been infected with COVID-19 worldwide, from almost all countries, with 4.6 million deaths, according to the WHO. Reflecting the danger of the pandemic, the WHO and the Center for Disease Control and Prevention "CDC" declared that strict precautionary measures should be applied by governments to combat the spread of the virus (Anderson et al., 2020). The WHO warned that successful control of the pandemic depends upon how and when governments apply such precautionary decisions. The importance for governments to balance strict precautionary measures with the negative impact on daily life activities and the economy has also been stressed (Shim et al., 2020).

The Kingdom of Saudi Arabia (KSA) is the largest country in the Arabian Gulf region, with an area of 2,150,000 km^2^, and about 34,218,169 residents, most of them under the age of 65. Immigrants make up 38.3% of the total population. The cites of Mecca and Madinah, in the western region of KSA, are the destination for millions of Muslim pilgrims each year, where they perform religious rites named “Hajj and Umrah” (Ebrahim and Memish, 2020a). The number of pilgrims traveling to Saudi Arabia has been increasing steadily annually, reaching about seven million in 2019 (Atique and Itumalla, 2020a). The annual management by the kingdom of Saudi Arabia of such a massive influx of people has provided the authorities with extensive expertise in health care surveillance and management during mass gatherings (Algaissi et al., 2020, Memish et al., 2014). In addition, the Saudi government has learned valuable lessons in dealing with previous health epidemics, especially the Middle East Respiratory Syndrome "MERS". MERS is caused by the coronavirus MERS-Cov that was first identified in KSA in 2012. It is a severe respiratory disease with high mortality, transmits from camels to humans, but person-to-person infection is also possible in health care settings (Assiri et al., 2013). The outbreak investigation procedures were designed in collaboration with Canadian expertise when they were applied, tested, and improved over time since 2012, and have demonstrated a high success rate (Assiri et al., 2013).

The strongest strategy adopted by the Saudi policymakers was early intervention and application of national mitigation measures before the first coronavirus case was detected in the country on 2^nd^ of March 2020. By January 2020, the Saudi Ministry of Health (Saudi MOH) was already disseminating information about the unknown virus, routes of transmission, and precautionary actions through television, radio, SMS text messaging and social media platforms in 12 languages. Clear instructions about the importance of handwashing and maintaining personal hygiene were clearly displayed in all public places and means of transport. In order to facilitate user's access to the health care services and their transfer between care types, the Ministry of Health (MOH) launched health clusters in all the Kingdom's 13 regions. A Health Cluster is an integrated network of health care providers (includes primary, secondary and tertiary care) under one administrative structure, serving a certain geographical area and allowing mobility of medical professionals within the health clusters system. The cluster played an important role in triaging and transferring Covid-19 patients to appropriate specialized care centers within regions.

## Managing the Pandemic in ten strategies

### Quarantine of epidemic areas, and travel restrictions

By 6 February 2020, before the travel ban, the Ministry of Education evacuated all Saudi students from countries known to be experiencing Coronavirus outbreaks, including Beijing and Hong Kong, UK and Italy. Immediately after the first case was reported in KSA on 2^nd^ March 2020, the MOH mandated all travelers arriving from a country where there was a coronavirus outbreak to disclose this fact to the authorities (in the interests of national health security). On arrival, all passengers and students, including asymptomatic ones, were hosted in hotels, shelter houses or schools. They were tested serologically for the virus at designated time intervals, for 14 days. All housing, food provision and test services were funded entirely by the government. Employees suspected of infection were directed to quarantine at home for at least 14 days and given sick leave. International flights were banned totally from early March 2020. When flights did resume on 17^th^ May 2021, travel entry into the Kingdom of Saudi Arabia was subject to following strict mandatory precautions and guideline for all foreign nationals (including Saudi Residents), that included quarantine and PCR testing (Arabnews, 2021b) .

Internally, as most cases were reported from the Qatif and Mecca regions, the government announced a temporary but strict city-wide quarantine (SPA, 2020b). Transportation between cities was suspended for 6 months, which minimized the spread of the epidemic. In February 2021, a partial lockdown was implemented in response to the global warning about the second wave of COVID-19 as well as the new strains reported from the UK and Africa. The Ministry of Interior announced monetary fines and/or detention for violators of orders such as curfews, and social distancing in public places.

### Expansion of serological screening

Proactive surveillance was implemented for people who had come into contact with any suspected COVID-19 cases. Previous National experience in dealing with epidemics of MERS-CoV coronavirus has undoubtedly helped the government in its efforts to train healthcare workers and to improve surveillance activities(Assiri et al., 2013). Field teams of public health specialists from the MOH responded promptly to the identification of a case, and investigating all their possible contacts, and reinforcing home quarantine. An early priority was the scaling up of active surveillance through mass screening on 21 April 2020, which started in high-risk areas such as crowded slums and densely populated worksites (MOH, 2020). From July 2020 onwards, around 320 primary health care centers or clinics (named “Tatamman”) were set up across the KSA to serve symptomatic individuals. These had a capacity of 30,000 visits per day. Also, 26 drive-through centers (named “Ta'akad”) were set up, with a capacity for 50,000 PCR tests per day, serving even asymptomatic visitors. The PCR results were returned through the mobile app “Sehaty” in a 12–24 hour period (Alghamdi et al., 2020). As of 8 September 2021, 27,65,314 serological screenings had been carried out.

### Mask wearing and social distancing

As of 5 March 2020, the Saudi government has taken further precautions by limiting entry to the two holy mosques (Mecca and Medinah). After Hajj, visitors were allowed to enter only when they showed both a time permit through the Tawakkalna App and no current infection of COVID-19 or vaccinated status. All these procedures were done electronically (ArabNews, 2021a). Governmental and private sector employees were directed to work from home until further notice, internal flights were suspended, and social gatherings of any kind, including parties, weddings, and conferences, were suspended. All mosques nationwide were closed. A lockdown was enforced gradually, starting with a nationwide curfew initially from 6 pm–7 am (Yezli and Khan, 2020). Then, high-risk areas, such as the areas surrounding the two holy mosques, had 24 hours a day curfews implemented. People residing in Mecca (the area with the highest number of cases) were subject to quarantine restrictions for three consecutive months. The areas of Riyadh, Jeddah and Medina were also under 24-hour curfews for 21 days. Exemptions to quarantine were available only for vital services such as home deliveries and healthcare.

As of 9 March 2020, schools and universities were suspended for the entire academic year 2020–2021, with the exception of some colleges that could put careful arrangements in place. The Ministry of Education made online education compulsory for all students (Ebrahim et al., 2020, Tanveer et al., 2020). However, implementing quality online education proved to be highly challenging. According to a study by Tanveer et al 2020 (Tanveer et al., 2020), some of the challenges reported included student worry and concern regarding assignments, little help from the tutors, difficulties in dealing with the learning management systems, fear of connection breakdown and graduation grades. Parents were put under pressure to provide computers and the internet for their families. Authors recommend that online education is an area for more in-depth investigation and improvement in KSA. By end of July 2021, the vaccine for 12-17 year-old children was made available and highly encouraged, which enabled most children to attend school physically by Sep 2021.

### Preparation of hospitals to deal with the influx of coronavirus cases

The high preparedness of the health care sector was a major factor in achieving a high rate of recovery, reaching up to 98%, and a low death rate of 0.7% at the peak time of May 2020. KSA government dedicated and equipped 25 hospitals across the kingdom to deal with the treatment of individuals diagnosed with COVID-19. The average ICU bed occupancy of Covid-10 patients in KSA was 30%. Hence, a total of 58,900 beds, including 2200 isolation and 9000 ICU beds with mechanical ventilation, were spared to take care of cases that needed hospitalization (Alqahtani et al., 2021). All health care sectors -without exceptions- were directed to notify positive cases through a unified Epidemiological Surveillance Program named «HESN». It provides surveillance of communicable diseases data to develop effective control programs by decision -makers. It also helped to detect, respond to, and monitor notifiable diseases; unify health processes, forms and reports across the Kingdom; and minimize surveillance disparities throughout different health affairs and facilities (MOH, 2019).

To increase healthcare capacity within hospitals, administrative precautions were taken, such as delaying elective surgeries and postponing non urgent outpatient appointments. Telemedicine, through the use of “virtual clinics” played a significant role. Moreover, the Saudi Center for Disease and Control (Saudi CDC) in cooperation with the MOH released several protocols for health care specialists about the management of COVID-19 patients: an isolation protocol, a hospital admission protocol, management of pregnant and cancer patients with COVID-19 protocol, guidelines for home quarantine, workplace and public place protocols, and many more (MOH-Publications, 2020). The use of these protocols was promoted through the MOH website and social media (in both Arabic and English l)– to be adopted by all health sectors within KSA. The MOH assigned a toll-free hotline (937) to provide immediate support to the public, answer questions, and receive notifications of suspected cases at all times 24/7. The appointment booking and individuals’ health information services were implemented through an application named (Sehaty). Health care professionals were made available to provide consultations and to transfer suspected cases to the nearest testing center.

Preparedness also included fast-track hiring of respiratory therapists and ICU nurses, and large trade deals with China and Korea to buy more diagnostic kits and mechanical ventilators. In addition, in collaboration with the private sector, the MOH established four field hospitals in high risk areas with a capacity of +1100 beds each, including laboratories, pharmacies, operating rooms, and digital scanners linked with all hospitals. Leaders from the MOH and volunteers from diverse medical specialties have assisted in serving in the field hospitals. Like every other country, there was an urgent demand for a supply of ICU beds and mechanical ventilators, but the government managed to meet this demand. Several national health-supplies factories joined to produce sterilizers, masks and ventilators which helped in the sustainability of medical equipment during the pandemic. It was established by data projection that there was no surge capacity for intensive care beds as long as the epidemic curve was kept flattened (Alqahtani et al., 2021).

### Using big data and Artificial Intelligence solutions

The Saudi government has been supporting ministries and other entities to move into digital services. However, during the pandemic, this effort was accelerated at the highest level with the demand for data integration. The Saudi Data and Artificial Intelligence Authority (SDAIA) developed the Tawakkalna App to support governmental efforts aimed at countering COVID-19. Initially, the app was developed to facilitate the issuance of travel permits electronically during the curfew period. Then it was developed to be a national database integrated with many governmental organizational systems and fed by real-time inputs and GPS data. In collaboration with the MOH, the app is being used to monitor individuals’ movements during quarantine, to give notifications to users when they have been in an area of positive cases, and to prove vaccination status (Hassounah et al., 2020) Recently, the app has been used to tissue restricted time permits for pilgrims visiting the two holy mosques and the Hajj.

Further, Saudi researchers have developed a digital tracking device for positive cases in the form of electronic bracelets. The bracelets have health sensors and linked to a real-time database and a mobile application called “Tatamman,” The Internet of Things IoT framework provides infrastructure communication for three devices using secure channels. This infrastructure enables the authorities to check the health of individuals and track their adherence to restrictions. The data enabled leaders to analyze and visualize possible risks and facilitate decision-making (Khalid;, 2020, Mukhtar et al., 2021). The MOH used digital screens that show real-time data with immediate processing inside the COVID-19 master control rooms in which specialists were tracking information such as the spread of the disease, admissions, and death rates, and then giving daily recommendations. These technologies helped the policymakers to take informed decisions with regard to the precautionary measures and strategies. Ultimately, in 2020 the kingdom achieved the 43th rank out of 193 countries in the E- government as per United Nations, the Division for Public Institutions and Digital Government. This ranking has improved by 9 points since 2018(Nations, 2021). This aligns with the kingdom's Saudi Vision 2030, one of the foci of which is investment in information and communications technology, including in the healthcare sector.

### Public assurance and fighting misinformation

It is known that in times of national crisis, fear and misinformation play an important role in worsening the situation and may contribute to misplaced governmental priorities (Jones, 2020). The MOH in cooperation with its collaborators (Ministry of Interior, Saudi CDC) used all forms of media (Radio, TV, social media, newspapers, text messaging) to communicate with the public the necessity for taking precautionary social distancing, hand and face hygiene steps and to take the vaccines. The MOH has sought to build trust with the public by holding daily press conferences about the epidemiological status of the outbreak, where they share timely statistics regarding Covid-19 related cases and deaths. There is an emphasis during these meetings to provide accurate information, and dispel myths and disinformation. On 19^th^ March 2020, King Salman "the custodian of the two holy mosques" gave a speech to the public, to reassure them that ensuring the health of the Saudi population was a priority for the government. That all people living in Saudi Arabia regardless of immigration status would receive free medical care and should consult without fear. Additionally, stocks of food were maintained at normal levels during the peak of the pandemic (Riaydhxpress, 2020). Social media influencers - especially those who were infected - have been contributing to the awareness and fighting the stigma of COVID-19 by sharing their stories. The Health Minister gave a speech on 5^th^ May 2020, assuring the public that the Kingdom is doing a good job in fighting the COVID_19 pandemic, with death rates ten times lower as compared to the worldwide rate, and the burden on the health care facilities is manageable where 96% of ICU beds are vacant. That speech delivered a great hope that the Kingdom would be successful in containing the disease(SPA, 2020a). In the context of community engagement, the MOH has recruited and trained over 32000 volunteers until August 2020, to raise awareness; help in hospitals, quarantine facilities, mass testing centres and laboratories; provide home care; deliver medications; and provide support services (Khan et al., 2021).

### Removing slum areas and re housing of its inhabitants

About 33% of the residents of Saudi Arabia are foreigners. Based on infection rates during April to June 2020, non-Saudis represented about 80% of the cases. The holy cities of Mecca and Madinah were the main sources of these cases; most cases were residing in large immigrant slums that had been present for decades. The majority of people living in these slums were residing in KSA illegally, and therefore had limited or no access to medical care. The crowded nature of the housing, and the lack of access to appropriate sanitary measures (running water, etc.) made these environments ideal for virus transmission between the inhabitants. Many residents also do not speak Arabic, which hampers the spread of information (Wasdani and Prasad, 2020). Because lockdown might not be enough in such contexts, the government has been putting in place strict actions to eradicate the slums and house their residents in appropriate accommodations until they test negative for the virus. As an incentive, in a move that is both intelligently self-interested and humanitarian, King Salman announced that the government would provide free treatment for all residents, including unauthorized immigrants (Nereim;, 2020).

### Cancelling of the Hajj season

Hajj Season is the largest annual mass gathering in the world. Around 3 million Muslim pilgrims from over 180 nations travel to the city of Mecca, KSA to perform the Hajj religious rites (over a period of 4–6 days). KSA has always taken the responsibility of hosting the pilgrims and assuring their safety as an honor and a duty. Hajj is a national priority for the government. From March 2020, the Hajj event has been suspended on the recommendation of health experts to prevent coronavirus transmission at home and abroad. The experience of previous infectious disease outbreaks in the past two decade (SARS-CoV Ebola virus, Zika virus, and MERS-CoV) helped health leaders in KSA to better prepare the country and its health systems to cope with the impact of Covid-19. By July 2020, the government announced that the Hajj season would be limited to a thousand internal pilgrims of specific age groups and health status (Ahmed and Memish, 2020, Ebrahim and Memish, 2020b). Exceptional efforts were made to apply social distancing at all times, and a high level of organization was applied to move the pilgrims through all the places of Hajj. These measures, in addition to home quarantine before and after the Hajj, the availability of free PPE for all pilgrims, and providing a health leader for each subgroup, served well to limit transmission. The handling of the Covid-19 crisis by KSA may be used as a model in how to best deal with infectious disease outbreaks and maintain a level of normality in day to day operations and social functions.(Atique and Itumalla, 2020b, Mahdi et al., 2020). The Umrah and Hajj Season in Mecca is a large source of income for the country and of international religious significance. The cancellation of the Hajj season demonstrates the government's commitment to maintain the health of the nation and how seriously they took the international warnings regarding Covid-19 despite the painful economic impact on the country.

### Safeguard the National Economy

It is well known that the Covid-19 pandemic has negatively affected the economies of all countries, especially in terms of high healthcare expenditure in screening for and treating Covid-19 cases. The mitigation measures to combat the pandemic have had serious effects on major pillars of the Saudi economy through decreased demand for oil and airlines services, disruption of religious tourism, and manufacturing functions and supply chains. The government allocated a stimulus package; an “Emergency budget” of US$ 32 billion, and provided other aid initiatives. Business owners were exempted for 3 months from value-added tax, excise tax, income tax (Finance, 2021). Two government bodies have initiated responses focused on overcoming the crisis with the least possible economic damage. First, the Saudi Arabian Monetary Authority (SAMA) initiated programs such as funds to support the private sector with up to US $5 billion. They postponed payments on debt, and provided many more bank packages to stabilize finance. Furthermore, the Ministry of Finance allotted up to US $9 billion to subsidize 60% of Saudi residents’ salaries, and US $40 billion to support economic activities in the private sector. In addition to increasing VAT to 15%, improving oil prices and the use of a strong banking sector are keys to the recovery of the economy, and to boosting growth opportunities (Abusaaq, 2020). Finally, the KSA government has supported scientific research development during the pandemic by providing funds, expertise and equipment for researchers. MOH, SCDC, and King Abdul Aziz City for Science and Technology (KACST), as well as many research centers and universities, have issued fast track research funds for COVID_19. The research priorities were diagnosis, treatment, and vaccine development. Recently, the Saudi National Health Institute was opened to act as an umbrella to support and fund all health related research in KSA (Khan et al., 2021).

### COVID-19 Vaccines campaigns

**Figure 1:**
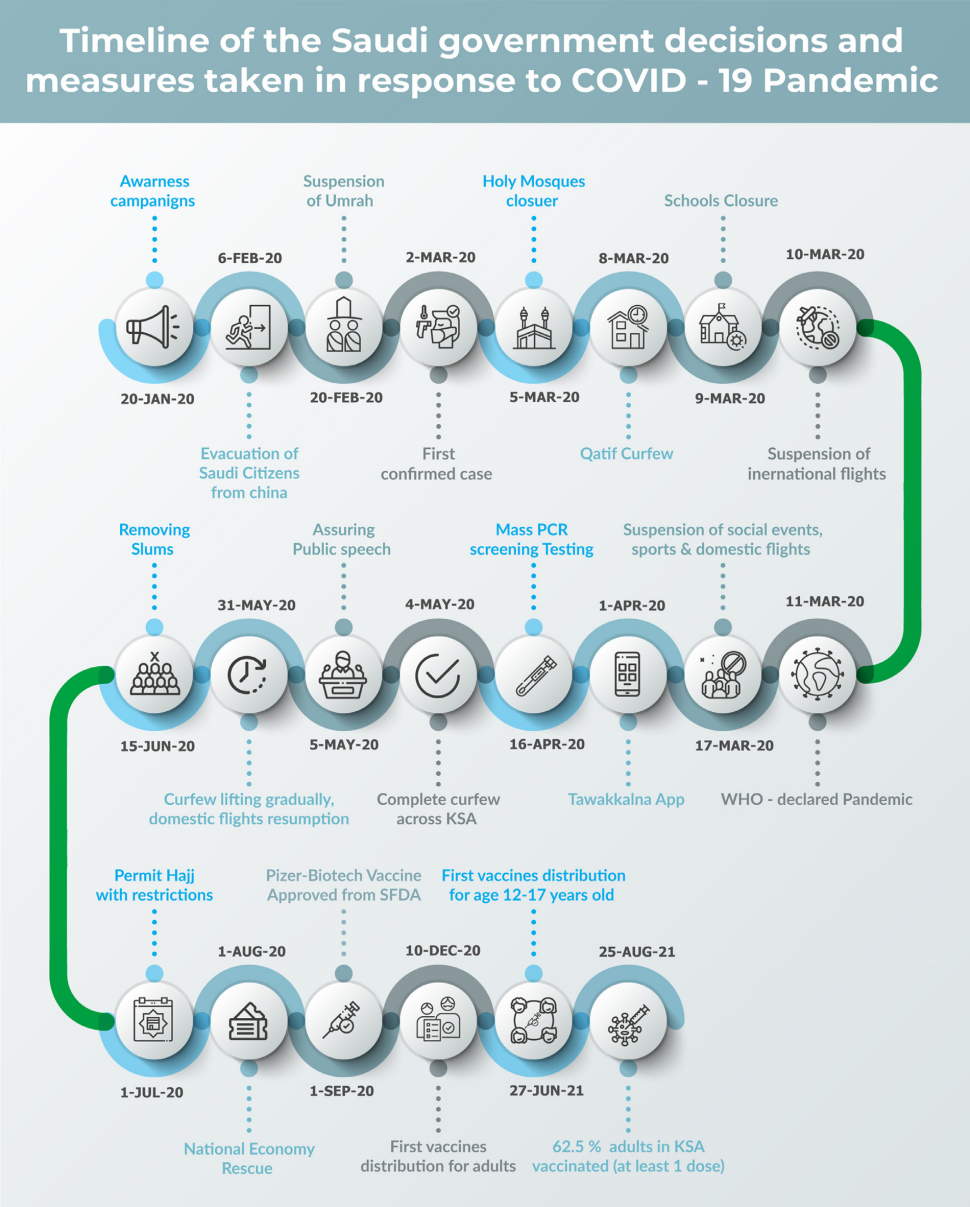
Timeline of the government decisions and measures taken to combat the COVID-19 Pandemic in Saudi Arabia as of September 2021.

Once the vaccine was approved by the WHO, KSA was among the first countries to authorize it for use in the country, and distributed it nationwide. It was available for free, and prioritized initially to high-risk populations and frontline medical staff, then for all citizens, residents and those living illegally within the country. A plan for vaccine distribution was periodically evaluated carefully to ensure fair and priority driven coverage. The vaccines used were: Oxford/AstraZeneca, Pfizer/BioNTech, and Moderna. Many national campaigns to raise awareness were conducted by the MOH, medical professionals and social media influencers (Barry and BaHammam, 2021). King Salman Bin Abdul Aziz and Crown Prince Mohammed Bin Salman received their vaccines early, the event was televised and disseminated via media to help increase vaccine uptake by the public. The vaccines were first available from December 2020 at publicized venues in four big cities, and were then rolled out in 587 primary health care centers across the kingdom. The vaccinations centers were well designed and offered high quality and exceptional customer experience. The turnaround time was 15 minutes, short waiting times and fast service encouraged more people to get vaccinated. As of January 2021 Saudi Arabia had secured the second-highest number of vaccines of all countries in the Middle East, after the UAE. Figure 1 shows a timeline of the government decisions taken to contain the COVID-19 Pandemic nationally.

## The outcome

**Figure 2:**
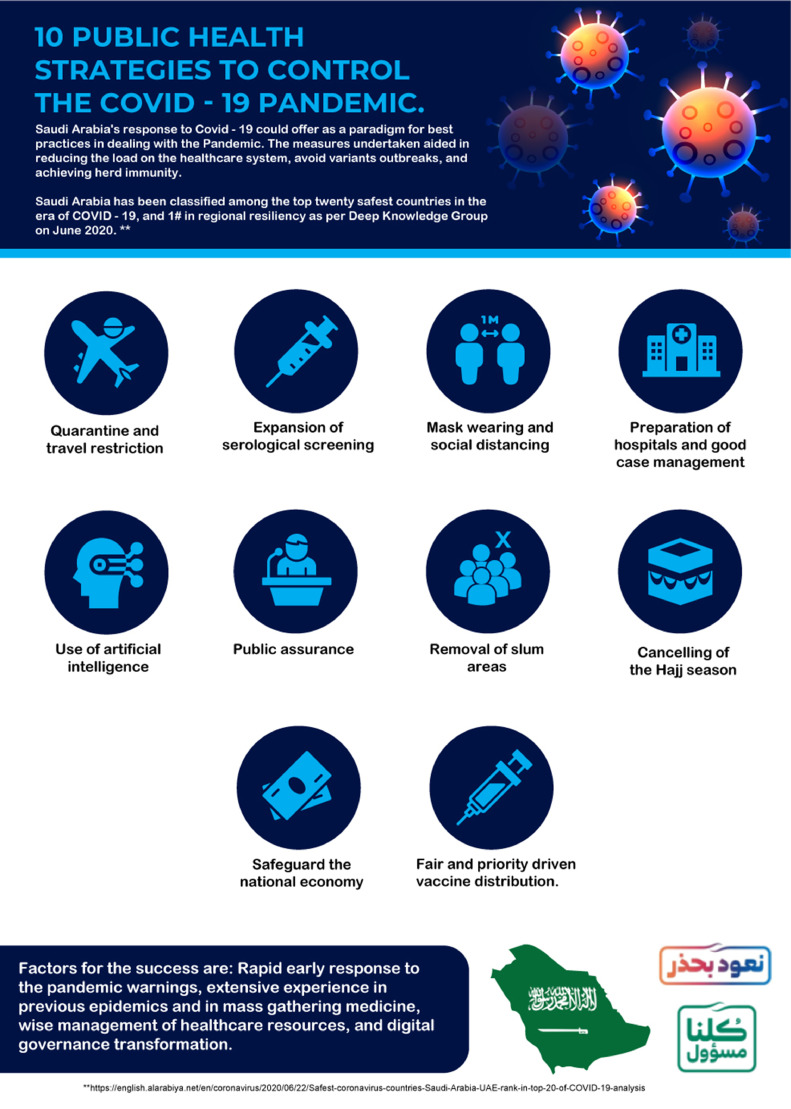
The Ten public health strategies of Saudi Arabia to control the COVID_19 pandemic successfully

The government of Saudi Arabia has dedicated every effort to control the COVID-19 pandemic, prioritizing the safety and health of its residents. Although the economic cost to the country ran into billions of dollars and affected the national economy, the government have been taking strong decisions in quick time, all to the interest of the residents; Figure 2 summarizes these efforts. Up until the time of writing this report, the following metrics help support the success of the implemented strategic measures the country took to contain the virus.

1 The infection rate is low, with 474 infected per million people compared to 620 in the US (Worldometer, 2020).
2 The recovery rate of COVID-19 cases has been rising dramatically, up to 96%, because of early diagnosis and management. The MOH reported that recovery cases were higher than new confirmed cases in many periods (MOHSaudi, 2020).
3 The case fatality rate is much lower than the international average (1.6% vs 2.2%). This reflects medical care, the availability of ICU beds and mechanical ventilators.
4 The high level of awareness of the public with respect to the danger of COVID-19 and the routes of infection, and its complications, especially on elderly people, helped to enforce effective precaution measures (Alahdal et al., 2020).
5 The hospitalization rate of COVID-19 cases is around 18%, and the rate of ICU bed occupancy is around 30%. Overall, hospitals did not have surge capacity for ICU beds (Alqahtani et al., 2021).
6 The multidisciplinary collaboration between intergovernmental bodies and international agencies was unprecedented, and fruitful (Hargreaves et al., 2020).
7 Saudi Arabia has been awarded the first rank among Arabic countries in scientific publications as per the Nature index and the 29^th^ rank in the world for the period from May 2020 until April 2021(Index, 2021).
8 By 21 August 2021, the vaccination rate is about 96.2 doses per hundred people (∼34.81 million). At this rate, Saudi Arabia could have 70% of people vaccinated (2 doses) in 58 days (or by Oct 18, 2021) (covidvax.live, 2021) .
9 Saudi Arabia ranked the second after China for the most optimistic country to recover economically within a year of COVID-19 Pandemic. The survey was done during the World Economic Forum between 25^th^ June to 9 July 2021, including 21,500 people from 29 countries(Forum, 2021).

**Figure 3:**
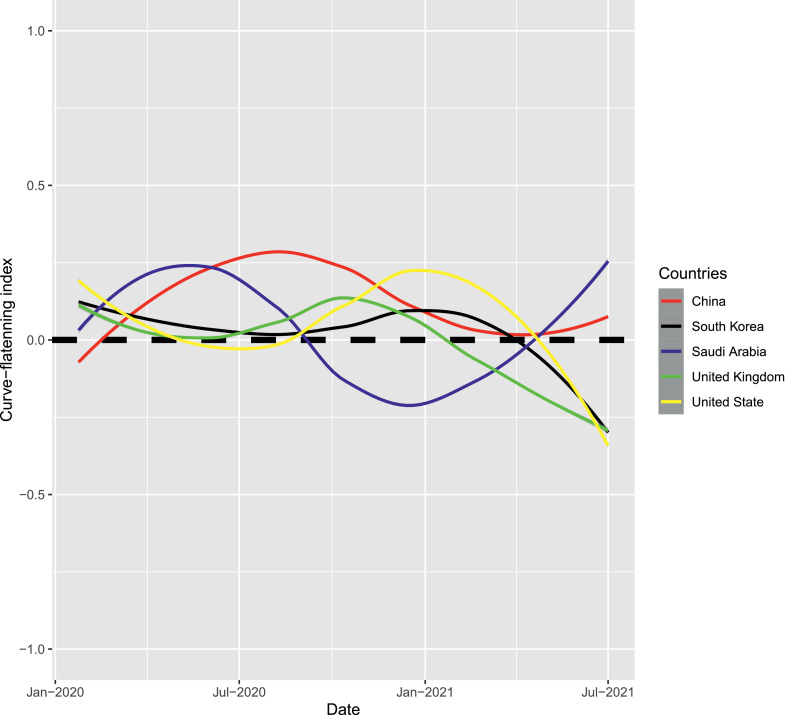
This is a measure of how well a country is flattening the epidemic curve at any point in time. It is a graphical illustration of an index (Ct)which is calculated as the change in growth rate divided by the magnitude of the growth rate, and then the whole multiplied by negative 1 so that positive values are “good” values meaning growth rates are declining at that point in time, and negative values are “bad”. It should rightly be thought of as an index of “suppression” rather than an index of “curve-flattening”. Retrieved from: https://drkhalid.shinyapps.io/COIVD19/ on 4 November 2020 @11:36

Despite mathematical prediction models for the COVID-19 infection rate in Saudi Arabia (Alrashed et al., 2020, Alzahrani et al., 2020), it has become obvious that Saudi Arabia is one of the few countries that has effectively dealt with the pandemic's social, political, economic, and, most crucially, health-care-related implications (Alanezi et al., 2020, Citizen, 2020). Figure 3 shows a comparison of the flattening of the curve in KSA with that in the United States, United Kingdom, South Korea, and China. A recent study comparing KSA mitigation measures with those of other countries supported this finding (Alanezi et al., 2020).

## Conclusion

The measures taken by Saudi Arabia in handling Covid-19 may serve as a model for best practice in dealing with the virus. The country adopted a “whole government” approach as recommended by the WHO in controlling the coronavirus pandemic of 2020. The comprehensive efforts, along with high levels of awareness regarding Covid-19, by the Saudi population helped limit the frequency and severity of cases and kept mortality rates from the virus low. Removing slums, the continuous need for healthcare supplies, and balancing the impact of Covid-19 restrictions while keeping the country running have been some of the biggest challenges the country has faced.

## Appendix Supplementary materials

Image, application 1
