# Supplementary material for: Ten public health strategies to control the Covid-19 pandemic: the Saudi Experience
Source: IJID Reg. 2021 Sep 17;1:12–9. doi: 10.1016/j.ijregi.2021.09.003 (PMC8447545; doi:10.1016/j.ijregi.2021.09.003)
Supplement: Supplementary file 1 [file mmc1.pdf]

AUTHORSHIP AGREEMENT

All requests for authorship changes after submission of a manuscript to *IJREGI* should be made to the Editorial Office. This form must be completed for any authorship changes including adding new authors, removing existing authors, reordering existing authors, or adjusting equal contributor status. The corresponding author must confirm that all authors meet the criteria for authorship as outlined by the Committee on Publication Ethics (COPE) and that all authors agree to the change. All authors who have been added, removed, or reordered need to confirm that they agree to the change by signing the form. Please complete and return this form. The relevant submission will be put on hold and no further processing can occur until the editorial office receives the completed form.

Manuscript number:

Manuscript title:

Would you like to change the authorship of your paper? (Please tick one of the following):

- o Yes (please fill in box below with all necessary signatures):

The nature of change(s) in authorship (Please check one of the following):

- o Change de Corresponding Author:
- o Add new author(s);
- o Remove existing author(s);
- o Change the order of authorship;
- o Others (please specify):

Detailed reason for the change:

Two authors (E.D and Y.T) preferred not to be included as IJID Regions journal is new.

Indicate the specific change:

Adding a new author

**Name**	Maliha Nasim
**Email address**	Maliha.nasim77@gmail.com
**Institution**	King Faisal Specialist Hospital and Research Center
**Specific contribution**	Maniscript language editing and scientific reviwing

Removing an existing author

**Name**	Edward De Vol, Yasmin AlTwaijri
**Email address**	edevol@kfshrc.edu.sa, yasmint@kfshrc.edu.sa

**Institution**	King Faisal Specialist Hospital and Research Center
**Agree to be acknowledged**	Yes Yes

Complete author order AFTER change (please note any authors with equal contribution as first authors or corresponding authors)

Order	Author name BEFORE change	Author name AFTER change	Signature
1	Areej AlFattani	Areej AlFattani	
2	Amani AlMeharish	Amani AlMeharish	Amani
3	Edward De Vol,	Maliha Nasim	M. Nasim
4	Yasmin AlTwaijri	Khalid AlQhatani	
5	Khalid AlQhatani	Sami AlMudraa	
6	Sami AlMudraa		
7			
8			
9			
10			

Approval of the final version of the manuscript to be submitted, all authors must sign the table above to indicate agreement with the altered authorship (all authors must be listed, full name in print and signatures are needed from all).
